# Trends in Computer-Assisted Surgery for Total Knee Arthroplasty in Germany: An Analysis Based on the Operative Procedure Classification System between 2010 to 2021

**DOI:** 10.3390/jcm12020549

**Published:** 2023-01-09

**Authors:** Tizian Heinz, Annette Eidmann, Philip Anderson, Manuel Weißenberger, Axel Jakuscheit, Maximilian Rudert, Ioannis Stratos

**Affiliations:** Department of Orthopaedic Surgery, Koenig-Ludwig-Haus, Julius-Maximilians University Wuerzburg, Brettreichstrasse 11, 97074 Wuerzburg, Germany

**Keywords:** robotic, TKA, knee replacement, computer navigation, Germany

## Abstract

Alignment strategies for primary total knee arthroplasty (TKA) have changed significantly over time with a shift towards a more individualized alignment goal. At the same time, computer-assisted surgery (CAS) has gained interest for intraoperative control and accuracy in implant positioning and limb alignment. Despite the often discussed benefits and drawbacks of robotics and navigation for TKA, the routine use of these new devices on a day-to-day basis remains obscure. Therefore, nationwide hospital billing data based on the Operation Procedure Classification System (OPS) were retrieved from the Federal Statistical Office of Germany for the period from 2010 to 2021. OPS codes for primary total knee arthroplasty (OPS code: 5-822*) were further analyzed regarding the usage of computer navigation (additional OPS code: 5-988) or robotic devices (additional OPS code: 5-987). Gender and age at the time of surgery were also assessed. The results show a total of 2,226,559 primary TKAs were implanted between 2010 and 2021, of which 2,044,914 were performed conventionally (91.84% of all TKAs). A total of 170,276 TKAs were performed using navigation technique (7.65% of all TKAs) and another 11,369 TKAs were performed using robotics (0.51% of all TKAs). For the period from 2018 to 2021, a substantial increase in robot-assisted TKA (R-TKA) was observed, with an average increase rate of 84.74% per year, while the number of navigated TKAs declined (−3.67% per year). Computer-assisted surgery, and particularly robotics for TKA, are seeing growing popularity and stepwise translation into routine clinical use in Germany, with a steep increase rate of more than 80% per year since 2018. Nevertheless, the majority of TKAs are still performed using manual instrumentation, rendering conventional TKA the currently unchanged gold standard.

## 1. Introduction

Total knee replacement surgery has been proven to be effective for the end-stage osteoarthritis of the knee joint. According to the German institution for quality control and transparency in health services (IQTIG), it is performed more than 152,000 times every year [1]. Furthermore, the trajectory of primary knee arthroplasty is predicted to experience a steep increase during the next 10 years, reaching a volume of more than 220,000 primary TKAs every year [2]. At the same time, patient dissatisfaction after TKA remains high [3,4]. This has led to the questioning of long-held concepts associated with TKA ranging from the design of the prothesis to implant materials, and more recently focused on the alignment of the prothesis, to enhance patient satisfaction and functional outcomes. The current interest in implant orientation seems to be bifold, with efforts for increasing the precision and accuracy of well-established alignment concepts on the one hand, and the development of novel patient-specific and more individualized alignment strategies (i.e., kinematic alignment, functional alignment, restricted kinematic alignment) on the other hand. Technology, and especially computer-assisted surgery (CAS), have proven suitable for achieving unrivalled precision and accuracy in implant orientation and, combined with the merits of real-time intraoperative feedback on implant positioning, this has in turn eased the development of novel implant strategies [5,6,7].

Since the first introduction of CAS for TKA in 1997, major advances have been achieved both in technology as well as in implant designs, materials and biomechanical concepts [8,9]. Traditionally, CAS has been used for increasing technical precision and the realization of immaculate mechanical alignment of the prothesis. While several studies were able to demonstrate higher precision regarding the implant position with CAS compared to manual instrumentation, clinical outcome scores did not necessarily reflect improvement, which has led to the routine use of CAS for TKA being questioned [10,11]. With the recent development of individualized alignment strategies, CAS has regained interest as a method of intraoperative alignment control allowing for real-time feedback in terms of restoring native knee anatomy during TKA implantation. Especially the alignment concept of TKA has seen interest boosted over the last few years, promoted by several studies showing promising results based on the kinematic alignment concept. Though being achievable with manual instrumentation by the measured resection technique, CAS has eased the way for individualized alignment strategies in a streamlined manner. The advent of imageless robotic systems has further eased the routine use by sparing patients time-consuming preoperative diagnostics like CT or MRI.

According to Muir et al., CAS can be classified into three different systems [12]: (1) passive systems, (2) hybrid systems and (3) active systems. In passive systems, saw jig orientation is checked using the computer system, but bone cuts are still made manually and bone cut orientation is determined by the surgeon. Passive systems are traditionally considered as the basic form of computer navigation. With hybrid systems, the surgeon follows a predefined and intraoperatively determined cut orientation, typically with the use of robotic arms, but the bone cut is still performed by the surgeon. With active systems, both the cut orientation and the bone cut itself are performed by the computer system through robotic arms.

While computer navigation and robotics for TKA are consistently reported in the scientific literature, the degree to which their translation and integration into routine clinical use has progressed is mainly unknown. Therefore, the aim of this study was to highlight past and current practice trends of CAS for primary TKA based on clinical data of the German “Operation and Procedure Classification System” (OPS) retrieved from the Federal Statistical Department of Germany.

## 2. Materials and Methods

### 2.1. Source Data

All data were retrieved from the Federal Statistical Office of Germany (https://www-genesis.destatis.de (accessed on 3 September 2022)) using the data set 23141-0103 (title: “Age groups, patient’s place of residence, surgeries, and surgical procedures”). This data set includes all patient-related billing data for every hospital in Germany. The data set 23141-0103 was filtered by the Federal Statistical Office of Germany to include patients who were coded with the surgical OPS code 5-822* (5-822.0 to 5-822y; codes used for implantation of a TKA) with or without the complementary code 5-987 (code used for application of a surgical robot) or 5-988 (code used for application of a navigation system) for the years 2010 to 2021. From this period, 2,226,559 TKA were identified and embedded in the analysis. Additional data from the data set included gender (male; female) and patients’ age at the time of surgery (grouped in 5-year periods). The data were requested and were sent to us on 30 September 2022. All source data can be downloaded from the URL https://github.com/ioannis-stratos/G-DRG_Robotic (generated and accessed on 20 November 2020).

### 2.2. Operation and Procedure Classification System (OPS)

The OPS was introduced in Germany in January 1995 as a mandatory classification system for all kinds of surgical procedures. The German OPS is based on the international classification of procedures in medicine (ICPM). After its first introduction in 1995, the OPS had been revised three times by the year 2004, and since then a new and upgraded version has been released every year. The OPS holds about 7800 different classifiable procedures. The additional codes for the use of a surgical robot (OPS 5-987) or a navigation system (OPS 5-988) for TKA have been included since 2001.

### 2.3. Groups

Using the filtered dataset provided by the Federal Statistical Office of Germany, we defined three groups: a conventional group (i.e., patients who were coded for TKA without the supplementary codes for application of a surgical robot or application of a navigation system); a navigated group (i.e., patients who were coded for TKA with the supplementary code for application of a navigation system); and a robotic group (i.e., patients who were coded for TKA with the supplementary code for application of a surgical robot).

### 2.4. Data Processing

Data sets were rearranged from tabular form to list form. Systematic data analysis was performed using Tableau (Tableau Software; Seattle, United States). Linear regression analyses were chosen as the statistical analysis method to show correlations over time. Nonlinear (Gaussian) regression analyses were used to calculate mean values and standard deviation for the factor age. To compare groups, one- or two-way ANOVA (depending on the number of the factors) was used. Statistical analysis for regression analysis was performed using an F-test. All tests were calculated with GraphPad Prism v.9 (GraphPad Software; San Diego, CA, USA). Values are given as absolute values or mean ± standard deviation. The exact significance level is stated in the legend or the text.

## 3. Results

**Figure 1:**
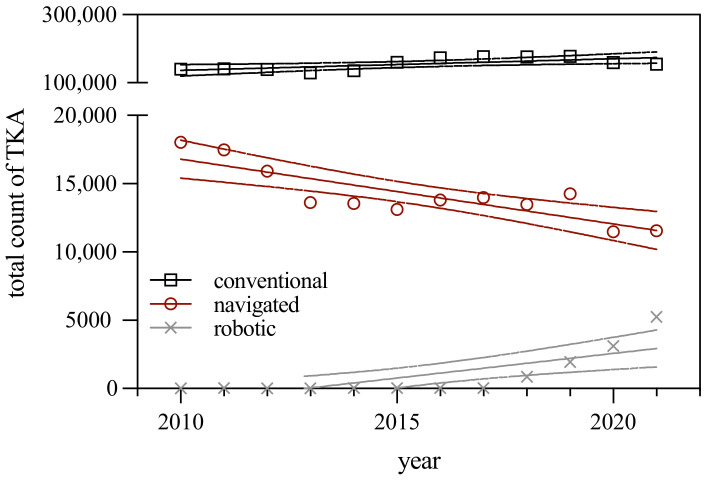
Total number of three different types of TKA (conventional, navigated and robotic) performed in Germany between 2010 and 2021. Illustrated are the regression lines as well as the 95% confidence intervals. Complementary data for the regression lines are given in Table 1.

Between 2010 and 2021, we could identify for the German population 2,044,914 conventionally operated TKAs (91.84% of all TKAs), 170,276 navigated TKAs (7.65% of all TKAs) and 11,369 robotic-assisted TKAs (0.51% of all TKAs). The number of conventionally operated TKAs increased over time with an average increase rate of 1.19% per year. In the same period, the number of navigated TKAs declined (−3.67% per year). From the year 2010 to the year 2017, only 23 robotic-assisted TKAs were documented annually on average. The robotic-assisted TKA was predominantly implemented in TKA surgery after the year 2018 with 863 documented cases. For the period 2018 to 2021, we could observe a substantial increase in robotic-assisted TKAs with an average increase rate of 84.74% per year (Figure 1 and Table 1).

The gender distribution between conventional TKA and navigated TKA was similar over the past 11 years (0.60♂:1♀ for conventional TKA vs. 0.61♂:1♀ for navigated TKA). Upon the introduction of robotic-assisted TKA, more male patients were treated using robots (0.92♂:1♀ for robotic TKA) compared to conventional TKA or navigated TKA.

**Figure 2:**
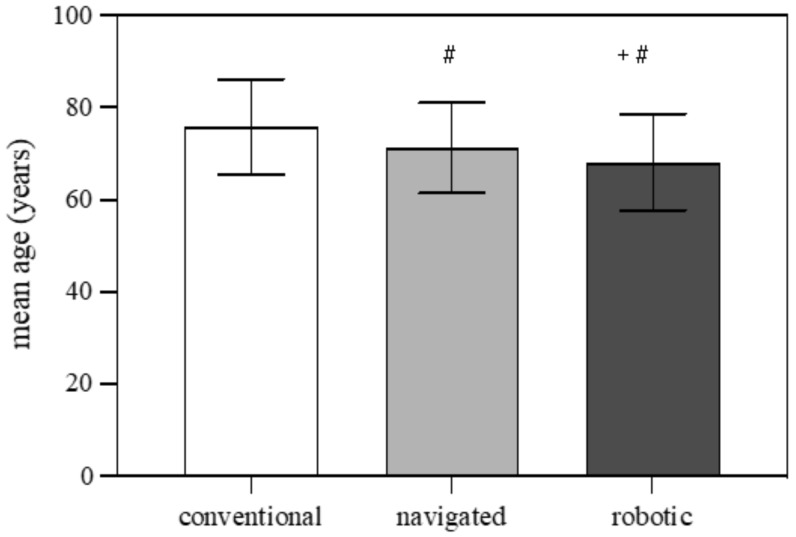
Bar chart for the mean age of the patients who were coded with three different types of TKA (conventional, navigated and robotic) performed in Germany between 2010 and 2021. Values are given as mean ± standard deviation. Two-way ANOVA; # *p* < 0.0001 vs. conventional; + *p* < 0.0001 vs. navigated.

Patients who underwent a robotic-assisted TKA were on average younger compared to navigated TKA patients, and they in turn were even younger compared to conventionally operated TKA patients (75.77 ± 10.33 years for conventional TKA vs. 71.17 ± 9.86 years for navigated TKA vs. 68.08 ± 10.47 years for robotic-assisted TKA; Figure 2). Further subgroup analysis for gender showed that male patients were on average one to two years younger than female patients for all groups (conventional TKA, navigated TKA and robotic-assisted TKA; Table 2).

## 4. Discussion

Although total knee replacement surgery has shown satisfactory long-term results, with revision rates below 5% ten years after implantation, the efforts and ambition to optimize patient satisfaction, functional outcome and implant longevity continues, especially in the light of a relatively constant dissatisfaction rate of up to 20% following TKA [13,14,15,16]. To date, the reason for this relatively high patient dissatisfaction still remains elusive, and attempts at improvement have been made both with altered implant designs (e.g., femoral single radius, multi radius, gradient decreasing radius, medial pivot) and alignment strategies (e.g., mechanical, kinematic, functional alignment) [17,18,19,20]. Inaccuracy of intraoperative implant orientation has been responsible for unfavorable outcomes and, consequently, tools for improved accuracy and precision have been developed. Both patient-specific instrumentation (PSI) and CAS have been shown to improve implant positioning and to produce fewer outliers [21,22,23,24]. However, increased accuracy has not necessarily translated into better functional outcome scores, thereby bringing into question the superiority of CAS and PSI over manual instrumentation. Especially in the light of the increased costs and operation time associated with CAS, surgeons’ attitudes towards CAS are often found to be ambiguous.

As a result of this study, the conventional manually instrumented TKA has to be still considered the current gold standard in Germany, with only about 8% of primary TKAs between 2010 and 2021 being implanted using computer-assisted technology. However, with the recent and steep increase in robot-assisted TKAs (R-TKAs), a substantial paradigm shift towards R-TKA is perceptible in Germany with an average annual increase rate of 84.74% since 2018. This shift towards R-TKA is well in line with recently published data from national registries worldwide. The Australian National Joint Replacement Registry has documented a steady increase in R-TKA since the year 2016, resulting in a total amount of nearly 20% R-TKAs by the year of 2020 [25]. The Norwegian joint registry has also documented a constant rate of 7% to 12% of R-TKAs since the year of 2007 [26]. Unfortunately, so far no other nationwide registries specify the explicit use of computer-assisted tools for the implantation of the prosthesis, hindering the evaluation of usage patterns of CAS for TKA. Boylan et al. showed a growth in computer-assisted TKA from 4.3% to 11.6% between 2008 and 2015 based on the Medicare Severity Diagnosis Related Groups of the State of New York [27]. Similar usage rates of CAS for TKA have also been reported for the United States by Bendich et al. [28]. By screening the billing data of 1,307,411 elective TKAs between 2010 and 2018, the authors documented a ratio of 92.8% conventional and 7.7% CAS TKAs. They were able to show the highest increase in R-TKA between 2016 and 2018. Overall, R-TKA shows a similar distribution throughout the western nations, with an ongoing implementation into the everyday practice of orthopaedic surgeons. However, clinical integration of R-TKA on a regular basis has still not been reached, as only 25% of 3330 surveyed surgeons stated that they use R-TKA in more than 75% of their cases [29].

The reasons for the steep increase in, and elevated demand for, R-TKA within the last five to six years seem to be multifactorial. While many studies in the past were able to show higher precision and better radiographic results with computer-navigated surgery, clinical results were usually not on par with the improved radiographic findings [30,31,32,33,34,35]. However, with the onset of robot-assisted surgery as the next evolutionary step in conventional navigation techniques, the technology has been refined and substantial advancements have been made. Because with conventional navigation techniques it has only been possible to passively monitor the alignment and implant positioning, the remaining risk of over- or mal-resection has not been overcome, leading to failure in some cases. Contrary to passive navigation techniques, semi-active robot systems actively assist the surgeon during bone resection, minimizing errors and boosting the overall precision of the TKA. Furthermore, robotic systems are capable of generating a virtual plan for the implant position during surgery, based on the pre- or intraoperatively obtained anatomy and soft tissue information about the knee, thereby allowing a more individualized component positioning with fewer soft tissue releases [36,37]. Song et al. demonstrated decreased extension–flexion gap imbalance when using R-TKA compared to conventional instrumentation [38]. Furthermore, clinical outcomes tend to be significantly better if the CAS implemented soft tissue balancing compared to computer systems not considering soft tissue balance [35]. Knee laxity has also been shown to be significantly higher in conventional TKA compared to R-TKA considering intraoperative soft tissue balancing [39]. Furthermore, several studies were able to demonstrate the superiority of R-TKA compared to conventional computer navigation in terms of surgery time, accuracy and length of hospitalization [40,41]. This may be the main reason for declining interest in the early computer navigation techniques in favor of new robotic systems which also factor in intraoperative soft tissue balance. Based on the German operative procedure classification, there was an average reduction every year for the computer navigation technique of 3.67% between 2010 and 2021.

Another reason for the high demand for R-TKA may be based on new alignment techniques which are hardly achievable without intraoperative visual control by computer systems. Recently, the functional alignment technique has been proposed as a new evolution of the kinematic concept. Software-based gap balancing and soft tissue balancing by modern robotic systems allow a more precise implant positioning, reducing the total amount of soft tissue release during TKA [42,43].

Despite the promising results of R-TKA, there are also some concerns associated with the regular usage of robotic surgery, as many authors and opponents of robotic TKA cite increased surgery times and costs [44,45]. However, several studies have demonstrated a relatively short learning curve regarding the duration of surgery, with similar operation room time for R-TKA compared to manual instrumentation after 10 to 20 cases [46,47,48]. In addition, R-TKA has also been associated with decreased overall blood loss, better clinical and radiographic outcome scores, as well as decreased length of hospital stays [49,50,51].

In view of the numerous reported benefits and the increased interest in R-TKA, it was the aim of this study to explore how the increased attention to R-TKA would translate into everyday clinical practice. Batailler et al. demonstrated that the evolving computer-based technology focusing on knee arthroplasty is not following the Scott Parabola, thereby setting CAS clearly apart from being only a temporary trend or fashion [52]. Unfortunately, to date, only the Norwegian and Australian joint registries are recording the usage of computer or robotic systems for TKA, which makes follow-up-, survival- and outcome-analysis after R-TKA easy. The German endoprothesis register (EPRD) does not record the use of computer or robotic systems for TKA. Such a record would be highly useful for the validation of the data presented here.

This is the first study that analyzes the application of CAS and robotics for TKA in German hospitals by using the German “Operation Procedure Classification System” (OPS). The total amount of more than two million primary total knee replacements analyzed for the last eleven years adds extra value to this study. Moreover, the German OPS differentiates between robotic surgery and computer navigation for TKA, which made it possible to clearly track the development and usage patterns of both systems during the last decade.

However, as the OPS is not linked to clinical data or results, it is impossible to keep track of revision rates and clinical outcome data. Moreover, as the OPS codes for computer navigation and robotic surgery only represent adjuvant codes, it is possible that not all TKAs with CAS have been accurately captured. Therefore, incorporating the kind of systems and devices used for TKA (manual instrumentation, computer navigation, robotics) into existing national and international joint registries would add great scientific value to the existing literature and help for further survival analysis and improvements in the field of modern, computer-assisted TKA.

## 5. Conclusions

Robot-assisted TKA is seeing high popularity and translation into routine clinical use in Germany with a steep increase rate of more than 80% per year since 2018. Simultaneously, computer navigation techniques are declining. However, the majority of TKAs are still performed with manual instrumentation, and therefore conventional TKA currently remains the gold standard. Further studies will be necessary to elucidate the long-term outcome of the ongoing global trend towards robot-assisted total knee replacement.

## Figures and Tables

**Table 1:** Regression analysis for three different types of TKA (conventional, navigated and robotic) performed in Germany between 2010 and 2021 (complementary table to Figure 1). The level of significance is shown in the last column (*p*-value) and demonstrates if each slope is significantly non-zero.

TKA Type	Equation	R^2^	*p*-Value
Conventional	y = 3407 x − 6,712,332	0.4671	0.0143
Navigated	y = −474 x + 970,241	0.7087	0.0006
Robotic	y = 361 x − 726,514	0.5992	0.0031

**Table 2:** Tabular chart from the mean age of patients who had a TKA between 2010 and 2021 in Germany divided by gender and the TKA surgical technique. Values are given as mean ± standard deviation. Two-way ANOVA; * *p* < 0.0001 vs. male.

TKA Type	Male (Age in Years)	Female (Age in Years)
Conventional	74.56 ± 10.56	76.50 ± 10.50 *
Navigated	70.05 ± 10.20	71.85 ± 9.56 *
Robotic	67.24 ± 10.03	68.89 ± 10.73 *

## Data Availability

All source data can be downloaded from the URL https://github.com/ioannis-stratos/G-DRG_Robotic (generated and accessed on 20 November 2020).
